# Psychiatric treatment considerations with direct acting antivirals in hepatitis C

**DOI:** 10.1186/1471-230X-13-86

**Published:** 2013-05-14

**Authors:** Sanjeev Sockalingam, Alice Tseng, Pierre Giguere, David Wong

**Affiliations:** 1 University Health Network, Program in Medical Psychiatry, Toronto General Hospital, 200 Elizabeth Street 8EN-228, Toronto, ON M5G 2C4, Canada; 2 Department of Psychiatry, University of Toronto, Toronto, ON, Canada; 3 University Health Network, Immunodeficiency Clinic, Toronto, ON, Canada; 4 Faculty of Pharmacy, University of Toronto, Toronto, ON, Canada; 5 The Ottawa Hospital, Immunodeficiency Clinic, Ottawa, ON, Canada; 6 The Ottawa Hospital Research institute, Ottawa, ON, Canada; 7 Toronto Western Hospital Liver Centre, Toronto, ON, Canada; 8 Faculty of Medicine, University of Toronto, Toronto, ON, Canada

**Keywords:** Hepatitis C, Mental disorders, Psychotropic drugs, Boceprevir, Telaprevir

## Abstract

**Background:**

Despite recent advances in hepatitis C (HCV) treatment, specifically the addition of direct acting antivirals (DAAs), pegylated interferon-alpha remains the backbone of HCV therapy. Therefore, the impact of DAAs on the management of co-morbid psychiatric illness and neuropsychiatric sequalae remains an ongoing concern during HCV therapy. This paper provides a review of the neuropsychiatric adverse effects of DAAs and drug-drug interactions (DDIs) between DAAs and psychiatric medications.

**Methods:**

We conducted a Pubmed search using relevant search terms and hand searched reference lists of related review articles. In addition, we searched abstracts for major hepatology conferences and contacted respective pharmaceutical companies for additional studies.

**Results:**

Limited data is available on the neuropsychiatric adverse effects of DAAs; however, data from major clinical trials suggest that DAAs have minimal neuropsychiatric risk. DAAs can potentially interact with a variety of psychotropic agents via cytochrome P450 and p-glycoprotein interactions. Triazolam, oral midazolam, St. John’s Wort, carbamazepine and pimozide, are contraindicated with DAAs. DDIs between DAAs and antidepressants, anxiolytics, hypnotics, mood stabilizers, antipsychotics and treatments for opioid dependence are summarized.

**Conclusions:**

Although DAAs do not add significant neuropsychiatric risk, the potential for DDIs is high. Consideration of DDIs is paramount to improving medication adherence and mitigating adverse effects during HCV therapy.

## Background

Treatment of hepatitis C virus (HCV), a virus infecting over 170 million worldwide [1], has evolved over the last two decades and moved from interferon-alpha monotherapy to pegylated interferon-alpha (IFNα) in combination with ribavirin therapy. HCV therapy with IFNα and ribavirin has yielded overall sustained virological response (SVR) rates of approximately 54% to 56% with SVR rates for genotype 1 approximating 45% to 50% [2,3]. The next generation of HCV therapeutic agents is direct acting antivirals (DAAs) that still require the use of interferon-ribavirin combination therapy. Protease inhibitors, specifically telaprevir or boceprevir, in combination with IFNα and ribavirin (i.e. triple therapy) have improved SVR rates to 70% to 75% in HCV genotype 1 patients [4,5].

Despite these enhanced SVR rates, psychiatric illness remains a barrier to widespread HCV treatment uptake due to the neuropsychiatric risks associated with IFNα. It is estimated that up to 50% of patients with untreated chronic HCV suffer from psychiatric illness when substance abuse and dependence is excluded [6,7]. Lifetime rates of mood, anxiety and personality disorders in untreated HCV-infected patients have each ranged from approximately 20% to 40% [6,7]. Treatment with pegylated interferon-alpha (IFNα) therapy can induce a myriad of neuropsychiatric side effects including depression in approximately 25% to 30% of patients undergoing IFNα therapy for HCV [8-11]. In addition, HCV-infected patients with pre-existing psychiatric disorders may experience an exacerbation of psychopathology secondary to IFNα.

Poorly managed psychiatric illness can lead to treatment discontinuation, poor adherence to treatment and serious psychiatric sequalae, such as suicide [12,13]. The onset of suicidal ideation and suicide on HCV therapy coincides with the onset of IFNα-induced depression (IFNα-D) and requires prompt recognition and treatment to prevent these serious psychiatric sequelae [12,14]. Integrated Hepatology-Psychiatric care models have demonstrated the capacity to mitigate neuropsychiatric risks associated with HCV therapy through improved access to psychiatric and psychological interventions [15,16].

In the era of DAAs, adherence is paramount to treatment success given the strict dosing regimen of first generation HCV protease inhibitors (PIs). First generation DAAs have high pill burdens and frequent dosing intervals. Active depression has been associated with poor antiviral therapy (ART) in patients infected with human immunodeficiency virus (HIV) [17]. Therefore, it is possible that poorly controlled psychiatric illness may compromise adherence to PI dosing schedules and as a result, reduce HCV treatment efficacy. Similar to the advent of HIV ART, first generation DAAs have also presented concerns regarding drug-drug interactions (DDIs) with medications including several psychotropic medications. Given the high prevalence of psychiatric illness in HCV-infected patients and need for psychotropic treatments for IFNα-induced neuropsychiatric side effects, an understanding of salient DDIs involving psychotropic medications is essential to the clinical care of patients treated for HCV.

With respect to DDIs, both boceprevir and telaprevir are substrates and inhibitors of CYP3A4 [18,19]. Both agents also inhibit p-glycoprotein [18,19] and telaprevir may inhibit renal transporters [20]. Approximately 50% to 60% of available prescription medications are metabolized via CYP3A4 pathway [21,22]. Moreover, preliminary HCV data suggests that in clinical practice, 72% of patients had at least one DDI and 50% had at least two DDIs related to DAAs [23]. Therefore, there is a high potential for DDIs with HCV protease inhibitors, particularly if treatment for other comorbid conditions is necessary.

Interactions may be pharmacokinetic or pharmacodynamic in nature. Pharmacodynamic interactions impact drug efficacy or toxicity in an additive, synergistic or antagonistic manner. For instance, pegylated interferon and ribavirin have CNS effects that overlap with those of the antiretroviral regimens involving efavirenz; co-administration may theoretically contribute to adverse effects including depression, mood changes, and suicidality. Clinicians may therefore wish to avoid this combination if possible, particularly in patients with a history of significant mental illness.

Pharmacokinetic interactions may result in altered concentrations of one or more interacting drugs. Negative two-way interactions have been observed between both boceprevir and telaprevir and ritonavir-boosted HIV protease inhibitors, with significant reductions in exposures of HCV agents and HIV protease inhibitors; therefore, telaprevir should not be coadministered with ritonavir-boosted darunavir, fosamprenavir, or lopinavir [18] and boceprevir is not recommended for use with any boosted protease inhibitor [24].

Negative consequences of drug interactions may include viral breakthrough and development of resistance, sub-optimal disease/symptom management, or drug toxicity and possible non-adherence [25]. These interactions highlight the challenges of managing multiple comorbidities in patients with HCV infection.

The purpose of this review was to evaluate the current evidence on: (i) the neuropsychiatric adverse effects of DAAs, and (ii) the DDIs between DAAs and psychotropic agents when used in HCV patients.

## Methods

We performed a Pubmed search using MeSH headings “hepatitis C” AND “boceprevir” OR “telaprevir” combined with “mental disorders”, “psychotropic drugs” and “drug interactions”. We limited our search to English language studies published between 2000-April 2013. References for all review articles were searched for additional studies as well as conference abstracts. Additional information on psychiatric adverse effects and DDIs with DAAs were requested from Vertex and Merck. Due to the limited literature, data on psychiatric adverse effects was also obtained from registration trials for boceprevir and telaprevir. Theoretical drug interactions were included in the respective sections. Due to available data on antidepressant efficacy in depressed HCV populations, we discussed potential DAA and antidepressant DDIs in the context of clinical evidence for specific antidepressant agents for treating depression during HCV therapy. Level of evidence was derived from 2 recent guidelines and existing reviews [26-29] and a previously published grading system [30] was used classify evidence for only studies examining antidepressant treatment of depression during HCV therapy.

## Results

### Neuropsychiatric side effects of DAAs

Data on neuropsychiatric adverse effects of DAAs is limited and predominantly derived from landmark clinical trials for boceprevir and telaprevir (see Table 1) [4,5,31-34]. Across trials, there was no significant difference in neuropsychiatric side effects between DAAs and treatment with peg- IFNα and ribavirin alone. It should be noted that the rates of neuropsychiatric sequalae from DAAs may be an underestimate, as patients with significant psychiatric illness were excluded from these studies and detection of psychiatric side effects did not utilize formal psycho-diagnostic tools. Only one study published data on anxiety during triple therapy and found a comparable reported rate of anxiety in patients treated with triple therapy (10%) versus standard therapy alone (12%) [4]. Although studies focusing specifically on psychiatric complications of DAAs are lacking, this preliminary data suggests that DAAs confer a minimal risk of additional neuropsychiatric side effects.

**Table 1:** Psychiatric adverse effects in DAAs

		**Telaprevir trials**			**Boceprevir trials**	
	**ADVANCE[**[4]**]**	ILLUMINATE*[[31]**]**	**REALIZE[**[32]**]**	**SPRINT-1[**[33]**]**	**SPRINT-2[**[5]**]**	**RESPOND-2[**[34]**]**
Psychiatric side effect						
Fatigue	57% (57%)	68%	55% (40%)	68% (55%)	53% (60%)	54% (50%)
Insomnia	32% (31%)	31%	26% (26%)	28% (38%)	33% (32%)	30% (20%)
Irritability	22% (18%)	-	14% (16%)	-	22% (24%)	19% (13%)
Depression	18% (22%)	-	9% (14%)	-	23% (22%)	12% (15%)
Anxiety	10% (12%)	-	-	-	-	-

% - percent for study arm corresponding to current standard of care for DAA.

(%) – percent for pegylated IFNα and Ribavirin treatment arm.

*ILLUMINATE – did not have pegylated IFNα and Ribavirin treatment arm.

### Antidepressant use with DAAs

Antidepressants are used primarily in the treatment of depression and anxiety in both untreated HCV patients and patients undergoing IFNα therapy for HCV. Studies have explored the use of antidepressants in HCV as both prophylactic (i.e. antidepressant pre-treatment) and symptomatic treatment for IFNα-D. Two recent guidelines have specifically identified management of IFNα-D and provided recommendations for antidepressant therapy in HCV-infected patients (see Table 2). Based upon these guidelines and previous reviews [26], only escitalopram currently has Level 1 evidence for treating or preventing depression emerging during HCV treatment [35,36].

**Table 2:** Evidence for antidepressant treatment of depression during HCV Therapy and drug interactions with DAAs

**Level of evidence for depression treatment**	**Antidepressant (route of metabolism)**	**Known or potential interactions with DAAs**	**Comments**
Level 1	Escitalopram (CYP2C19, 3A4 >> 2D6)	No interaction observed with boceprevir [37] 35% ↓ escitalopram AUC with telaprevir [38]	Boceprevir: no dose adjustment required. Telaprevir: May need to titrate escitalopram dose according to clinical response.
Level 2	Citalopram (CYP2C19, 3A4 >> 2D6)	Potential for ↓ antidepressant concentrations based on escitalopram interaction data.	Monitor and titrate dose according to clinical response.
	Paroxetine* (CYP2D6)	No interaction expected based on known pharmacologic characteristics.	Monitor and titrate dose according to clinical response.
Level 4	Bupropion (CYP2B6), Fluoxetine (CYP2D6)	No interaction expected based on known pharmacologic characteristics.	Monitor and titrate dose according to clinical response.
	Sertraline (CYP2B6 > 2C9/19, 3A4, 2D6, UGT1A1 - possible), Mirtazapine (CYP2D6, 1A2, 3A4), Venlafaxine (CYP2D6 > CYP3A4)	Potential for ↑ sertraline, mirtazapine, venlafaxine concentrations (clinical significance unknown).	Use with caution; monitor and titrate dose according to clinical response.
	Desvenlafaxine (UGT>>3A4) [39,40]	Potential for ↑ desvenlafaxine concentrations (clinical significance unknown).	Monitor and titrate antidepressant dose according to clinical response.
	Tricyclic antidepressants i.e. Desipramine (CYP2D6>>UGT), Imipramine (CYP2D6, 1A2, 2C19, 3A > UGT), Trazodone** (CYP2D6> CYP3A)	Potential increase in TCA concentrations resulting in dizziness, hypotension and syncope.	Use with caution with DAAs, lower TCA doses are recommended.
	Nortriptyline (CYP2D6)	No interaction expected based on known pharmacologic characteristics.	Monitor and titrate dose according to clinical response.
Avoid (exceptional circumstances only)	Duloxetine (CYP1A2, 2D6)	Duloxetine: risk of hepatotoxicity.	Duloxetine is contraindicated in liver disease.
	Nefazodone (CYP3A4)	Nefazodone: potential for ↑ nefazodone and/or DAA concentrations; also risk of hepatotoxicity.	Nefazone was discontinued in the United States and Canada in 2003 due to hepatotoxicity concerns. Avoid use in liver disease.
	St. John’s Wort (hypericum perforatum); induces CYP3A4 and P-gp [40].	Potential for ↓ DAA concentrations.	St. John’s Wort is contraindicated with boceprevir [19] and telaprevir [18].

*Evidence in RCT for depressed mood component of major depression only.

**Trazodone is primarily used clinically for treating insomnia.

**Level of Evidence:** Level I (≥ 2 RCTs or meta-analysis), Level 2 (1 RCT), Level 4 (Case reports/series or expert opinion).

Anxiety secondary to IFNα can also be treated with antidepressants, which are a first line treatment based upon the limited available literature (Level 4) [41,42]. Escitalopram and citalopram may be beneficial options in treating anxiety disorders in HCV based upon the anecdotal reports of safety in HCV [43-45] and extrapolation of evidence from non-HCV anxiety treatment guidelines [46]. Clinicians should be aware of the potential risk of dose-related QT prolongation with citalopram and escitalopram [47]. The maximum recommended dose is citalopram 20 mg per day in patients with hepatic impairment, those 65 years of age or older, patients who are CYP2C19 poor metabolizers, or patients who are taking concomitant cimetidine or another CYP2C19 inhibitor [48]. In some countries, such as Canada, the maximum recommended dose for escitalopram in patients with hepatic impairment is 10 mg per day due to QT prolongation concerns [49].

Drug interactions between DAAs and some antidepressants, specifically those affected by CYP 450 interactions of PIs, may lead to clinically significant adverse effects which impact tolerability to therapy for HCV. For example, SSRIs and Selective Noradrenergic Reuptake Inhibitors (SNRIs) can be associated with nausea, gastrointestinal upset, sweating and sexual dysfunction, which could emerge with PI related drug interactions.

Specific drug interactions with antidepressants and DAAs are summarized in Table 2. In a single study involving telaprevir, escitalopram area under the curve (AUC) was reduced by 35%, suggesting the need for clinicians to monitor the need for dose optimization on triple therapy [50]. No significant DDI has been observed between escitalopram and boceprevir [37]. Specific antidepressants, for example trazodone, that have a high sedative potential and potential for DDIs with DAAs can lead to increased sedation and may impact overall tolerability and compliance to both agents. Therefore, the selection of antidepressant agents during HCV therapy should include consideration of potential DDIs, in order to avoid possible adverse effects, which may negatively affect HCV antiviral treatment adherence. Clinicians should also be aware that St. John’s Wort is a potent inducer of CYP3A4 and P-gp [40], and is contraindicated with DAAs due to the potential risk for significant reductions in boceprevir or telaprevir concentrations [18,19].

### Benzodiazepine and hypnotic use with DAAs

Benzodiazepines may be a treatment option for anxiety symptoms in the context of HCV or secondary to IFNα; however, no large trials have examined the efficacy of anxiolytics in HCV [16,41,42,51,52]. Anecdotally, benzodiazepines have also been used short-term for insomnia in HCV-infected patients [41]. Furthermore, the prevalence of substance dependence in HCV patients has cautioned the use of benzodiazepines in this patient population. In general, short-acting benzodiazepines should be avoided due to potential rebound effect on anxiety and long-term benzodiazepine use may lead to tolerance and dependence.

If benzodiazepines are used, lorazepam, oxazepam or temazepam are preferred due to the reliance on glucuronidation, a process that is relatively preserved in patients with significant liver disease [53]. Furthermore, these three agents are the least susceptible to pharmacokinetic interactions with DAAs since they are not metabolized through the cytochrome P450 system. Most other benzodiazepine agents undergo metabolism solely or partially through CYP3A4, and thus concentrations may be increased by DAAs via CYP3A4 inhibition. Triazolam and oral midazolam are contraindicated with boceprevir and telaprevir, due to hypothesized or documented significant interactions. When administered orally, midazolam exposures were increased 430% in the presence of boceprevir [54] and almost 9-fold in the presence of telaprevir [55]. Intravenous midazolam concentrations increased 3.4-fold when co-administered with telaprevir [55]. Thus, while intravenous midazolam is not absolutely contraindicated with PIs, it is recommended that this combination be administered with caution in a setting which allows for close clinical monitoring for prolonged sedation and/or respiratory depression, and that dose adjustment of intravenous midazolam should be considered [19].

Zolpidem is metabolized through a variety of CYP450 isozymes, including CYP3A, 2C9, 1A2, 2D6, and 2C19. In the presence of steady-state telaprevir, zolpidem exposures were unexpectedly reduced by 47% [56]. Close monitoring and dose titration of zolpidem is recommended if this agent is coadministered with telaprevir. Zopiclone is also metabolized predominantly by CYP3A4 and to a lesser degree by CYP2C8 and CYP2C9. Zopiclone concentrations may theoretically be increased by DAAs and require close monitoring. Most other benzodiazepines should be used cautiously in patients on DAAs. Clinicians may consider starting with a decreased benzodiazepine dose and monitoring for benzodiazepine-related toxicity, or selecting an alternate agent such as lorazepam, oxazepam or temazepam. Dose reductions are also recommended in patients with severe liver impairment as per product monographs [18,19].

### Anticonvulsant use with DAAs

Anticonvulsants can be used as mood stabilizers for new onset or de-stabilized bipolar disorder during IFNα therapy for HCV. Studies on the efficacy of anticonvulsants as moodstabilizers in HCV are limited to case reports and as a result, treatment often follows non-HCV bipolar treatment guidelines [30].

Lithium is a preferred moodstabilizer due its renal excretion and minimal dose adjustment in patients with HCV except in patients with shifting fluid balance resulting from decompensated cirrhosis [57]. Lithium has no known drug interactions with DAAs. Valproic acid has no significant DDIs with DAAs; however, valproic acid use in HCV has been limited by its purported risk of hepatotoxicity [58]. Nonetheless, in a study of patients with less severe HCV disease, elevations in alanine aminotransferase (ALT) were comparable between valproic acid and other psychotropic agents [59].

Amongst the remaining moodstabilizers, carbamazepine is contraindicated due to induction of cytochrome P450 3A4 and potential for decreasing boceprevir or telaprevir levels (see Table 3). Lamotrigine undergoes extensive metabolism by UDP-glucuronosyltransferase (UGT) 1A4 [60]. This metabolic pathway is not inhibited or induced by boceprevir or telaprevir. Lamotrigine has been associated with severe rash, including Steven’s Johnson rash. Given that DAAs, particularly telaprevir, have also been associated with severe rashes, it is recommended to use extra precautions if coadministration is required. Gabapentin and pregabalin are not effective moodstabilizers for bipolar disorder in monotherapy [61]; however, based upon data from non-HCV populations pregabalin and gabapentin can be efficacious in treating co-morbid generalized anxiety disorder (GAD) in HCV. Both pregabalin and gabapentin have no significant drug interactions with HCV triple therapy involving DAAs as they are both predominantly renally excreted. Table 3 provides a summary of anticonvulsant drug interactions with DAAs.

**Table 3:** Anticonvulsant drug interactions with DAAs

**Drug (route of metabolism)**	**Known or potential interactions with DAAs**	**Comments**
Lithium (renal)	No interaction expected based on known pharmacologic characteristics	Monitor and titrate dose according to clinical response and serum levels.
Valproic Acid, divalproex Parent: UGT (50%), minor CYP dependent oxidation pathway (<10%) Inhibitor of UGT,CYP2C9/19	No interaction expected based on known pharmacologic characteristics	Monitor and titrate dose according to clinical response and serum levels.
Carbamazepine Parent: CYP3A>> 2C8, 1A2 Inducer of CYP3A, 2C9, 2C19, UGT and possibly 1A2	Potential for ↓ DAAs concentrations	Carbamazepine is contraindicated with boceprevir [19] Co-administration of telaprevir with potent CYP3A4 inducers such as carbamazepine may lead to reduced DAA plasma concentrations and decreased efficacy [18] Carbamazepine clearance can also potentially be decreased [62]. Consider an alternate agent with non-inducing metabolic properties.
Oxcarbazepine Parent: UGT Inhibitor of CYPC19; Potent inducer of CYP3A4. Relative to carbamazepine, oxcarbazepine inducing effect is 54% lower [63]	Potential for ↓ DAAs concentrations	Co-administration of boceprevir and telaprevir with potent CYP3A4 inducers, may lead to reduced DAA plasma concentrations and decreased efficacy. Consider an alternate agent with non-inducing metabolic properties [64].
Lamotrigine (UGT)	No interaction expected based on known pharmacologic characteristics	Monitor and titrate dose according to clinical response.
Gabapentin (Renal)	No interaction expected based on known pharmacologic characteristics	Monitor and titrate dose according to clinical response.
Pregabalin (Renal)	No interaction expected based on known pharmacologic characteristics	Monitor and titrate dose according to clinical response.

### Antipsychotic use with DAAs

Antipsychotic medications can be used during HCV therapy to stabilize pre-existing mood or psychotic disorders in patients or to treat IFNα-induced mood or psychotic symptoms secondary. Patients with severe mental illness, such as schizophrenia [65,66] and bipolar disorder [67] have been shown to have higher rates of HCV compared to the general population and thus, it may not be uncommon to treat patients with HCV who are already treated with antipsychotic medications for severe mental illness. Albeit rare, antipsychotic medications may be used to treat de novo secondary to IFNα [68-72]. In addition, atypical antipsychotics can be used for mood stabilization and irritability emerging during HCV therapy [41,73,74].

Several DDIs and side effects should be considered when prescribing antipsychotic medication in the context of HCV triple therapy (see Table 4). Telaprevir and boceprevir may interact with antipsychotics prone to corrected QT (QTc) interval prolongation and elevations in plasma levels could increase QTc prolongation risk. As a result, pimozide, a conventional antipsychotic with a high propensity for QTc prolongation, is contraindicated when treating patients with boceprevir and telaprevir. Amongst the atypical antipsychotics, ziprasidone, which is metabolized by CYP 3A4, is associated with an increased QTc prolongation risk amongst novel antipsychotics [75]. Initiation of ziprasidone should include a baseline electrocardiogram (ECG) and this may need to be reassessed on triple therapy for HCV due to DDI.

**Table 4:** Antipsychotic drug interactions with DAAs

**Drug (route of metabolism)**	**Known or potential interactions with DAAs**	**Comments**
Aripiprazole (CYP3A4, 2D6)	Potential for ↑ aripiprazole concentrations	Use combination with caution, and monitor for aripiprazole-related toxicity (sedation, sinus tachycardia, nausea/vomiting, or dystonic reactions). Consider starting with a decreased aripiprazole dose or select an alternate agent.
Asenapine (UGT1A4, CYP1A2)	No interaction expected based on known pharmacologic characteristics	Monitor and titrate dose according to clinical response [76].
Clozapine (CYP1A2> 3A4,P-gp)	Potential for ↑ clozapine concentrations	Clozapine has a narrow therapeutic index. Use combination with caution, and monitor for clozapine-related toxicity (Bone marrow suppression, generalized seizures, severe sedation, confusion and delirium). Consider starting with a decreased clozapine dose or select an alternate agent. When available, clozapine therapeutic drug monitoring is recommended [77,78].
Olanzapine (CYP1A2, UGT,PGP>2D6)	No interaction expected based on known pharmacologic characteristics	Monitor and titrate dose according to clinical response.
Paliperidone Primarily renally excreted (59%); minor CYP dependant pathway (CYP3A4, PGP>2D6), but may not be clinically significant. Substrate and inhibitor of P-gp [79]	Potential for ↑ paliperidone concentrations	DAAs inhibit both CYP3A4 and P-gp, and clinically significant interaction, although unlikely, cannot be ruled out. Use combination with caution, and monitor for possible paliperidone-related toxicity.
Quetiapine (CYP3A4>2D6, P-gp)	Potential for ↑ quetiapine concentrations	Use combination with caution, and monitor for quetiapine-related toxicity (excessive sedation). Consider starting with a decreased quetiapine dose or select an alternate agent [80].
Risperidone (CYP2D6, P-gp>3A4)	Potential for ↑ risperidone concentrations	Unlike its active metabolite paliperidone, risperidone is primarily metabolized by CYP2D6. However, the elimination of paliperidone may be impaired. Use combination with caution, and monitor for possible risperidone-related toxicity.
Ziprasidone (CYP3A4>1A2) Minor CYP dependant pathway (33%) [78].	Potential for ↑ ziprasidone concentrations	Although clinically significant interaction unlikely, use combination with caution, and monitor for possible ziprasidone-related toxicity (QTc).

Several antipsychotics are metabolized via CYP3A4/5, which are inhibited by current DAAs. Sedating antipsychotics that are metabolized by CYP3A4, such as quetiapine, may be increased via DDIs secondary to DAAs and could result in more pronounced sedation that could hinder compliance with multiple daily dosing regimens of DAAs. Clozapine is also metabolized in part by CYP3A4 and clozapine levels should be monitored closely during HCV triple therapy as higher doses of clozapine have been associated with an increased adverse effects including seizures [81]. Treatment with clozapine is further complicated during HCV therapy due to additive theoretical risks of agranulocytosis and neutropenia related specifically to IFNα effects. Therefore, clozapine monitoring protocols may need to be adjusted due to this risk and vigilant follow-up monitoring for signs of infection is recommended [82].

Lastly, DAAs are known inhibitors of P-gp and many second generation antipsychotics are substrates of P-gp [83]. In theory, inhibition of P-gp may lead to increased exposure of the antipsychotic in the CSF, and may be associated with enhanced effectiveness or toxicity [79]. Despite the absence of documented metabolic drug interactions, caution is to be exercised with known substrates of P-gp (quetiapine, risperidone, olanzapine) and DAAs.

### Addictions agents with DAAs

Given the higher rates of substance dependence in HCV-infected patient populations compared to the general population [6], treatment of concurrent substance use disorders, either through harm reduction or abstinence based models, is an important component of pre-HCV therapy stabilization. To date, no studies have determined if the addition of DAAs to HCV treatment increased the risk of substance use relapse.

In some HCV-infected populations, methadone treatment is a core component of HCV treatment stabilization in patients at risk of opioid and polysubstance dependence [84,85]. Methadone is metabolized by CYP2C19 and 3A4. The coadministration of methadone and telaprevir was shown to result in a 21% decrease of the active enantiomer R-methadone exposure [86]. However, free concentrations of R-methadone were unaffected and therefore no dosage adjustment is necessary. Buprenorphine pharmacokinetics are not affected by telaprevir and is safe for coadministration [87]. Boceprevir was studied with methadone, buprenorphine and naloxone. Similar to telaprevir, boceprevir led to a 15% decrease of R-methadone exposure. No free methadone concentrations were performed. Boceprevir was also associated with an increase of naloxone and buprenorphine exposure by 19 and 33% respectively, which is considered to be clinically non-significant [88].

## Discussions

Psychiatric disorders are highly prevalent in patients infected with chronic HCV and until IFNα-free therapies for HCV emerge, it is evident that neuropsychiatric risks of HCV therapy continue to be a significant concern. This review provides further information on the impact of DAAs on the neuropsychiatric sequelae of HCV therapy and clarifies the potential for DDIs with psychotropic medications.

First, DAAs do not appear to confer additional neuropsychiatric risks to patients undergoing HCV triple therapy. However, the use of DAAs warrants careful recognition of potential DDIs with psychotropic agents and an analysis of whether psychotropic regimens should be changed due to significant DDI risks. In addition, the potential for DDIs with psychotropic agents may exacerbate side effects and may interfere with DAA compliance, thus reducing HCV treatment efficacy.

The potential for clinically significant and complex interactions between DAAs and psychotropic drug classes is high. Interactions are primarily pharmacokinetic in nature, and may result in increased or decreased exposures of either/both drug classes. Potential clinical consequences of such interactions may include increased toxicity or potential under dosing. In the case of DAAs, sub-therapeutic concentrations may lead to treatment failure and development of resistance. Whenever possible, non-essential medications should be discontinued for the duration of HCV treatment.

Steps to identifying and managing interactions include ensuring that medication records are up to date at each patient visit (i.e., medication reconciliation), use of a systematic approach to identify combinations of potential concern, consulting pertinent HCV drug interaction resources, and frequent patient monitoring. Other management options include altering dosing frequency or replacing one agent with another drug with lower interaction potential. Given the complexity of this field, clinicians are encouraged to consult with pharmacists or physicians with expertise in HCV pharmacology when managing drug therapy of co-infected patients.

The results of this review can be beneficial in informing the selection of psychotropic agents for common psychiatric presentations in HCV. Using self-report or clinician rated psychiatric scales to measure treatment response to pharmacotherapy can be beneficial in monitoring relapse following psychotropic dose adjustments due to DDIs. For example, both the Beck Depression Inventory-II [89] or Patient Health Questionnaire-9 [87] for depression have been used and validated in this patient population. Further, awareness and education of the entire interdisciplinary treatment team is important in order to assist with prompt recognition of psychiatric symptoms, appropriate selection of psychotropic agents with minimal drug interactions and to minimize adverse effects to increased overall treatment adherence. The importance of interdisciplinary models of HCV care is evident from studies showing comparable HCV treatment adherence rates and outcomes for patients with either active substance use [84,90] or severe mental illness [91,92] as compared to controls.

## Conclusions

In summary, this review summarizes the emerging body of evidence in this area but also acknowledges the remaining gaps in the literature. Studies utilizing more detailed psychiatric assessment tools during HCV treatment with DAAs are needed to increase our understanding of DAA related psychiatric complications. Additional drug interaction studies between DAAs and commonly used psychotropic agents are urgently needed. The results of these studies will be essential to guiding clinicians presented with challenges in interpreting DDI risks related to psychiatric care in the era of HCV triple therapy, in order to optimize HCV treatment outcomes and as well as management of psychiatric symptomatology.

## Competing interest

The authors have no funding interest to declare with respect to this study.

## Authors’ contribution

SS has served as a speaker for Roche Canada. AT has received honoraria for consulting work with Merck- Canada and Vertex Pharmaceuticals. PG has served as a speaker, a consultant and an advisory board member for Vertex Pharmaceuticals and Merck Frosst Canada. DKW has received nursing support from Roche Canada and Shering-Plough Canada. All authors read and approved the final manuscript.

## Pre-publication history

The pre-publication history for this paper can be accessed here:

http://www.biomedcentral.com/1471-230X/13/86/prepub
